# Phylogeography of Kyasanur Forest Disease virus in India (1957–2017) reveals evolution and spread in the Western Ghats region

**DOI:** 10.1038/s41598-020-58242-w

**Published:** 2020-02-06

**Authors:** Pragya D. Yadav, Savita Patil, Santoshkumar M. Jadhav, Dimpal A. Nyayanit, Vimal Kumar, Shilpi Jain, Jagadish Sampath, Devendra T. Mourya, Sarah S. Cherian

**Affiliations:** 1 Maximum Containment Laboratory, ICMR-National Institute of Virology, Sus Road, Pashan, Pune, 411021 India; 2 Bioinformatics Group, ICMR-National Institute of Virology, Pune, 411001 India

**Keywords:** Phylogeny, Molecular evolution

## Abstract

The Kyasanur Forest Disease (KFD) has become a major public health problem in the State of Karnataka, India where the disease was first identified and in Tamil Nadu, Maharashtra, Kerala, and Goa covering the Western Ghats region of India. The incidence of positive cases and distribution of the Kyasanur Forest Disease virus (KFDV) in different geographical regions raises the need to understand the evolution and spatiotemporal transmission dynamics. Phylogeography analysis based on 48 whole genomes (46 from this study) and additionally 28 E-gene sequences of KFDV isolated from different regions spanning the period 1957–2017 was thus undertaken. The mean evolutionary rates based the E-gene was marginally higher than that based on the whole genomes. A subgroup of KFDV strains (2006–2017) differing from the early Karnataka strains (1957–1972) by ~2.76% in their whole genomes and representing spread to different geographical areas diverged around 1980. Dispersal from Karnataka to Goa and Maharashtra was indicated. Maharashtra represented a new source for transmission of KFDV since ~2013. Significant evidence of adaptive evolution at site 123 A/T located in the vicinity of the envelope protein dimer interface may have functional implications. The findings indicate the need to curtail the spread of KFDV by surveillance measures and improved vaccination strategies.

## Introduction

Kyasanur Forest Disease (KFD) is a tick-borne viral hemorrhagic disease caused by the Kyasanur Forest Disease Virus (KFDV). The virus is a member of family *Flaviviridae*, genus *flavivirus* and categorized as a bio-safety class-4 level pathogen^1^. KFDV was first identified and isolated from sick and dead monkeys in the forest regions of Shimoga district situated in the State of Karnataka, India and reported during the year 1957^2^. Eventually, KFD infections were noticed among persons who visited the forests. Monkeys and humans are most susceptible to the KFDV, and the virus gets transmitted through hard ticks belonging to the genus *Haemaphysalis*^3^. Since the discovery of KFDV, the disease was investigated in and around Karnataka state and it was observed that the KFDV was reported from only five districts of Karnataka namely, Shimoga, Chikkamagalore, Uttara-Kannada, Dakshina-Kannada, and Udupi^4^. The case-fatality rate in humans was noted to be 2–10%^5–7^.

The KFDV has a positive-sense, single-stranded RNA genome which is approximately 11 kb in length and encodes a single 3416 aa polyprotein that is cleaved post-translationally into a total of three structural (capsid C, pre-membrane prM, which is the glycosylated precursor to a small transmembrane protein M and envelope E) and seven non-structural (NS1, NS2a, NS2b, NS3, NS4a, NS4b and NS5) proteins^8^. As in other flaviviruses, the E protein plays a major role in infection of the cells and also in the protective immune response following virus infection. The E protein forms dimers on the surface of mature virions at the physiological pH while the surface of the immature particles is covered with trimers of prM–E protein heterodimers^9^. The prM of immature particles prevent irreversible inactivation during transport of the virus through the acidic compartments of the *trans*-Golgi network^10^. The NS1 protein of flaviviruses including that of the Tick-Borne Encephalitis virus is known to possess immune evasive functions^11^, induce oxidative stress and activate antioxidant defence^12^. Among the other non-structural proteins of flaviviruses, the NS3 protein^13^ contains a serine-protease domain at its N terminus whose activity requires the membrane-bound NS2B protein cofactor and an ATP-driven helicase and RNA triphosphatase at its C-terminal end, while the NS5 protein^14^ is a key replication enzyme with both methyltransferase and RNA polymerase activities.

Recent studies demonstrate the existence of KFDV in the States neighboring Karnataka including Kerala, Tamil Nadu, Goa, and Maharashtra^4^, which indicates the prevalence or spread of the KFDV arena to the nearby geographical locations. During the period, December 2011 to March 2012, out of 215 KFD suspected human cases, 28% of subjects were confirmed for KFDV infection^15,16^. Further, during the year 2014, a KFD outbreak was identified in Thirthahalli, Karnataka^17^ and during the year 2015, another outbreak was investigated in Shimoga District, Karnataka^4^. A KFD outbreak was reported in Goa during 2015^18^ followed by its detection in Sindhudurg district, Maharashtra state, during 2016^19,20^. Initial confirmation of KFD outbreaks was noted in Dodamarg and Sawantwadi talukas of Sindhudurg district, where human serum, monkey autopsy, and tick samples were found to be positive. Further newer hot spots were detected in other talukas Kankavli, Kudal, and Vengurla of Sindhudurg district in the year 2016^20^ and *Yadav et al*. (*unpublished data*). During 2016, there was confirmation of KFD cases amongst cashew-nut workers returning from Sattari taluka, Goa to Belgaum, Karnataka^18^.

The repetitive occurrence of disease outbreaks is a serious public health problem. Moreover, identification of KFDV from a different location is indicative of its dispersal to nearby locations. Such a scenario points towards a need to investigate the dispersal pattern of the KFDV from and within these different geographical locations. Molecular clock and phylogeography studies would help to identify the timescales of evolution, the emergence of newer lineages, the probable source of virus in a particular location, and track the transmission pathways of KFDV. Only limited phylogenetic and clock analysis of KFDV isolates are available in literature^8,21^. Hence, we performed phylogeographic analysis of KFDV strains from different States of India to get a better understanding of the molecular epidemiology. In the present study, we sequenced representative genomes of both the earlier and the recent isolates of KFDV. The isolates were collected from different geographic locations, covering the first KFDV isolation in Karnataka and the affected neighboring districts, and from the other affected States of Goa, Tamil Nadu, and Maharashtra. The study included isolates over the timespan 1957 to 2017 and from different species including ticks, monkeys, and humans. The evolutionary changes in the genome were noted and adaptive evolution in the different genes was studied to genetically characterize the recently circulating KFD viruses while a Bayesian coalescent-based approach was used to understand the phylogeographic pattern of the distribution of KFDV. Additional E-gene sequences of viruses available from the State of Kerala were also included in a phylogeography study of an enlarged E-gene dataset. Such phylogeography studies for KFDV based on time-sampled isolates from the States of Karnataka, Maharashtra, Goa, Tamil Nadu and Kerala are important to understand the dispersal pattern of the virus within Karnataka and its spread to the other locations.

## Results

### Next-generation sequencing of KFD virus isolates

Though majority of the isolates were sequenced by the Sanger sequencing method, the NGS method was implemented for the sequencing of some of the recent isolates (Supplementary Table S1). In addition, we also undertook sequencing of a few isolates by the NGS method for which the sequence was also done by the Sanger sequencing method. Concurrence of sequence information was noted (data not shown). In the case of NGS, the reference mapping of the generated FASTQ file was performed to an already available KFDV genome, as the isolates were known to be KFD positive. In all the samples, a significant number of reads were identified to be of KFD viral genomic RNA. It was found that almost 4–65% of reads were of KFDV origin depending on the viral load and the quality of the RNA library (Supplementary Table S2). It was further noted that more KFDV reads were retrieved in the Tissue culture Fluid (TCF) as compared to KFDV reads from mice brain suspension which may be due to contamination with the host sequences (of mice origin).

### Phylogeny and diversity analysis

Phylogenetic analysis based on the 48 whole genome sequences revealed a major group with strong bootstrap support, consisting of the recent strains from 2006 to 2017. The older KFDV strains from 1957 to 1972 though different, did not form a homogeneous cluster. The overall nucleotide (*nt*) divergence within all the KFDV strains based on the full genome was 2.24% while that between the two groups was 2.76%. The overall amino acid (*aa*) divergence within all the KFDV strains based on the full genome was 0.75% while that between the two groups was 0.86%. The values of *nt/aa* divergence in different genes/proteins are provided in Table 1. The highest percent *nt* difference in between the two groups was noted in the capsid gene (3.27%), followed by the E gene (3.22%) and NS2 gene (2.91%). The highest percent *aa* difference in between the two groups was noted in the capsid protein (3.17%), followed by the prM (1.66%), and NS2 (1.4%) proteins. The *aa* substitutions delineating the two groups were C: N56S; prM: F130L and NS1: S271N, NS3: G221R (except in KA_MCL13H113_H_2013 and KA_121863_H_2012) (Supplementary Table S3). A comparison of the source species-wise divergence (Table 2) in the different genes revealed that maximum *nt* divergence (2.39%) was observed between human and tick species, followed by that between human and monkey (2.23%) and lowest between monkey and tick (2.06%). In all three species, the maximum *nt* differences were noted in the capsid gene followed by the E gene and NS2 gene respectively.

**Table 1:** Comparison of the nucleotide and amino acid divergence in the different gene regions and proteins of KFDV based on whole genome sequences (n = 48) during the different time frames.

Region/Gene	Length		Percent divergence within recent isolate (2006 to 2017)		Percent divergence within old isolates (1957 to 1972)		Percent divergence between recent and old isolates		Overall percent divergence		Gene-wise dN/dS
	Gene	Protein	Gene	Protein	Gene	Protein	Gene	Protein	Gene	Protein	
	(Nucleotide) (*nt*)	(Amino acid) (*aa*)									
Genome	10248	3416	2.24	0.77	0.37	0.3	2.76	0.86	2.24	0.75	—
C	351	117	2.98	2.26	0.35	0.66	3.27	3.17	2.79	2.47	0.1917
prM	492	164	2.36	1.52	0.22	0.28	2.46	1.66	2.15	1.44	0.1136
E	1488	496	2.65	0.48	0.44	0.31	3.22	0.53	2.63	0.48	0.0274
NS1	1059	353	2.08	0.75	0.4	0.4	2.7	1.18	2.15	0.9	0.0643
NS2	1083	361	2.52	1.24	0.44	0.53	2.91	1.4	2.44	1.23	0.0881
NS3	1863	621	2.05	0.56	0.3	0.27	2.41	0.6	2	0.54	0.0408
NS4	1203	401	2.21	0.65	0.4	0.32	2.8	0.57	2.25	0.57	0.0406
NS5	2709	903	1.99	0.62	0.35	0.13	2.68	0.57	2.09	0.54	0.0369

**Table 2:** Species-wise comparison of the nucleotide and amino acid divergence in the different gene regions and proteins of KFDV based on whole genome sequences (n = 48).

Region	Within host divergence						Between host divergence					
	Monkey (M)		Tick (T)		Human (H)		M vs T		M vs H		T vs H	
	Gene	Protein	Gene	Protein	Gene	Protein	Gene	Protein	Gene	Protein	Gene	Protein
Genome	2.04	0.68	1.90	0.54	2.77	0.79	2.06	0.63	2.23	0.78	2.39	0.79
C	2.84	2.62	2.48	2.46	2.69	2.20	2.86	2.77	2.69	2.40	3.00	2.66
prM	1.81	1.02	1.66	0.86	2.29	1.64	1.81	0.97	2.12	1.53	2.31	1.51
E	2.50	0.38	2.36	0.30	2.58	0.52	2.54	0.34	2.60	0.51	2.48	0.53
NS1	1.84	0.88	1.77	0.69	2.24	0.86	1.87	0.78	2.16	0.99	2.29	0.95
NS2	2.26	1.02	2.02	0.69	2.55	1.46	2.25	0.87	2.40	1.31	2.54	1.20
NS3	1.73	0.43	1.75	0.46	2.02	0.55	1.83	0.46	1.93	0.53	2.19	0.61
NS4	2.09	0.59	1.75	0.22	2.34	0.70	2.01	0.43	2.30	0.63	2.34	0.51
NS5	1.93	0.55	1.79	0.43	2.11	0.52	1.93	0.52	2.11	0.54	2.22	0.59

Consideration of the enlarged dataset of 76 E-gene sequences revealed that the overall *nt* and *aa* divergence was 2.61% and 0.33% respectively. Supplementary Table S4 enlists the mutations noted in the E-gene sequences.

### Selection pressure analysis

**Figure 1:**
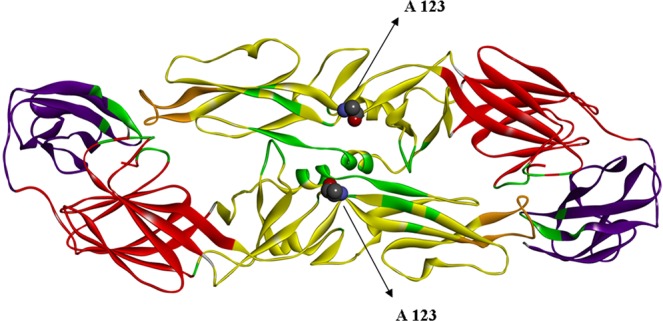
Homology model and positively selected site of KFDV E protein dimer: Homology model of E protein dimer of KFDV and mapping of the positively selected site 123 A. The E protein domains I, II and III are presented in red, yellow and violet, with the fusion loop in orange color. The dimer interface residues are shown in green color. The 123 A residue is displayed in a ball and stick representation in each monomeric unit.

The ratios of nonsynonymous to synonymous substitutions (dN/dS) determined for each KFDV gene (Table 1) showed no strong indication of positive selection. The selection pressure analysis (Table 3) based on 48 unique whole genome sequences, revealed few codon sites in prM: 152 V/I/A; E: 123 A/T; NS1: 271 N/R; NS2: 357 G/S/C and NS3: 14 R/G/T showing evidence of being positively selected. The dataset of 72 unique E-gene sequences also revealed the single site 123 A/T to be evolving under positive selection pressure. Among the sites, that showed evidence of positive selection pressure, prM: 152 V/I/A (269 in polyprotein numbering), falls in the envelope glycoprotein transmembrane region while E: 123 A/T (404) falls in the dimerization domain II of the glycoprotein^10^. Modeling of the KFDV glycoprotein was performed using the crystal structure of the Tick-borne encephalitis virus (TBEV), another tick borne virus belonging to the *Flaviviridae* family, available in the PDB (506 A.PDB)^9^. Mapping of the site revealed that *aa*123 was in close vicinity of the envelope dimer interface region (Fig. 1), that plays a crucial role during membrane fusion and infectivity^22^. No annotation of the NS1 protein of KFDV is available, and hence the site in NS1 that showed evidence of being positively selected, could not be functionally ascertained. In NS2, 357 G/S/C (1487) is noted to map on to the NS2B region while the NS3 site 14 R/G/T (1505) falls in the serine protease domain.

**Table 3:** Selection pressure analysis of KFDV isolates using the methods (SLAC, FEL, REL and MEME) available in the Datamonkey server.

Amino acid position in genome	Gene	Amino acid position in gene	MH 1721699 M 2017 (variable *aa’s)*	SLAC p-value	FEL p-value	REL Bayes Factor	MEME p-value	FUBAR Post. Pr.
4	C	4	G(R)	0.654	0.314	1.78	0.093	0.482
12	C	12	G(A)	0.963	0.554	0.284	0.095	0.088
22	C	22	T(P)	0.667	0.374	1.707	0.015	0.446
55	C	55	R(T)	0.707	0.472	1.461	0.052	0.444
118	PrM	1	A(V)	0.667	0.516	1.345	0.071	0.421
**269**	**PrM**	**152**	V(I,A)	0.297	**0.076**	**383.175**	**0.065**	0.883
**404**	**E**	**123**	A(T)	0.132	**0.037**	**109952**	**0.087**	**0.984**
710	E	429	M(V,I)	0.752	0.552	**153.495**	0.524	0.719
763	E	482	I(T)	0.904	0.743	0.1	**0.069**	0.215
**1048**	**NS1**	**271**	S(N,R)	0.359	**0.087**	**469.982**	**0.007**	0.886
1228	NS2	98	K(V)	0.589	0.386	38.482	**0**	0.72
**1487**	**NS2**	**357**	G(S,C,D)	0.298	0.044	705.118	**0.024**	0.939
1494	NS3	3	L(V)	0.889	0.795	0.093	**0.073**	0.219
1495	NS3	4	V(G)	0.963	0.274	0.139	**0.058**	0.063
1496	NS3	5	F(S,V)	0.504	0.259	5.036	**0.024**	0.617
1501	NS3	10	T(G)	0.84	0.59	4.893	**0**	0.194
1504	NS3	13	E(G,A)	0.916	0.335	14.811	**0.016**	0.078
**1505**	**NS3**	**14**	R(G,T)	0.351	0.208	**459.606**	**0.057**	0.875
1712	NS3	221	G(R)	0.328	0.18	**526.797**	0.199	0.892
1726	NS3	235	K(R)	0.731	0.502	1.427	**0.086**	0.438
1842	NS3	351	R(K)	0.686	0.363	1.653	**0.076**	0.464
1892	NS3	401	L(P)	0.378	0.26	2.022	**0.094**	0.498
2018	NS3	527	L(K)	0.282	0.398	16.093	**0**	0.678
2145	NS4	33	E(A)	0.729	0.472	1.503	**0.052**	0.449
2186	NS4	74	R(I)	0.687	0.668	1.088	**0**	0.427
2188	NS4	76	S(F)	0.845	0.093	0.171	**0.032**	0.068
2190	NS4	78	S(N)	0.505	0.161	13.335	**0.098**	0.689
2193	NS4	81	F(I,C)	0.505	0.259	5.032	**0.043**	0.617
2253	NS4	141	F(L)	0.71	0.358	1.867	**0.095**	0.448
2268	NS4	156	D(E)	0.673	0.521	**458.545**	0.67	0.665
2474	NS4	362	T(S)	0.667	0.313	2.034	**0.092**	0.468
2875	NS5	362	T(P)	0.667	0.313	2.034	**0.092**	0.468

Sites identified to be under positive selection pressure based on the statistically significance level* (shown in bold font) by at least two of the methods are shown in bold.

### Phylogeography and migration patterns

#### Based on the whole genome dataset

**Figure 2:**
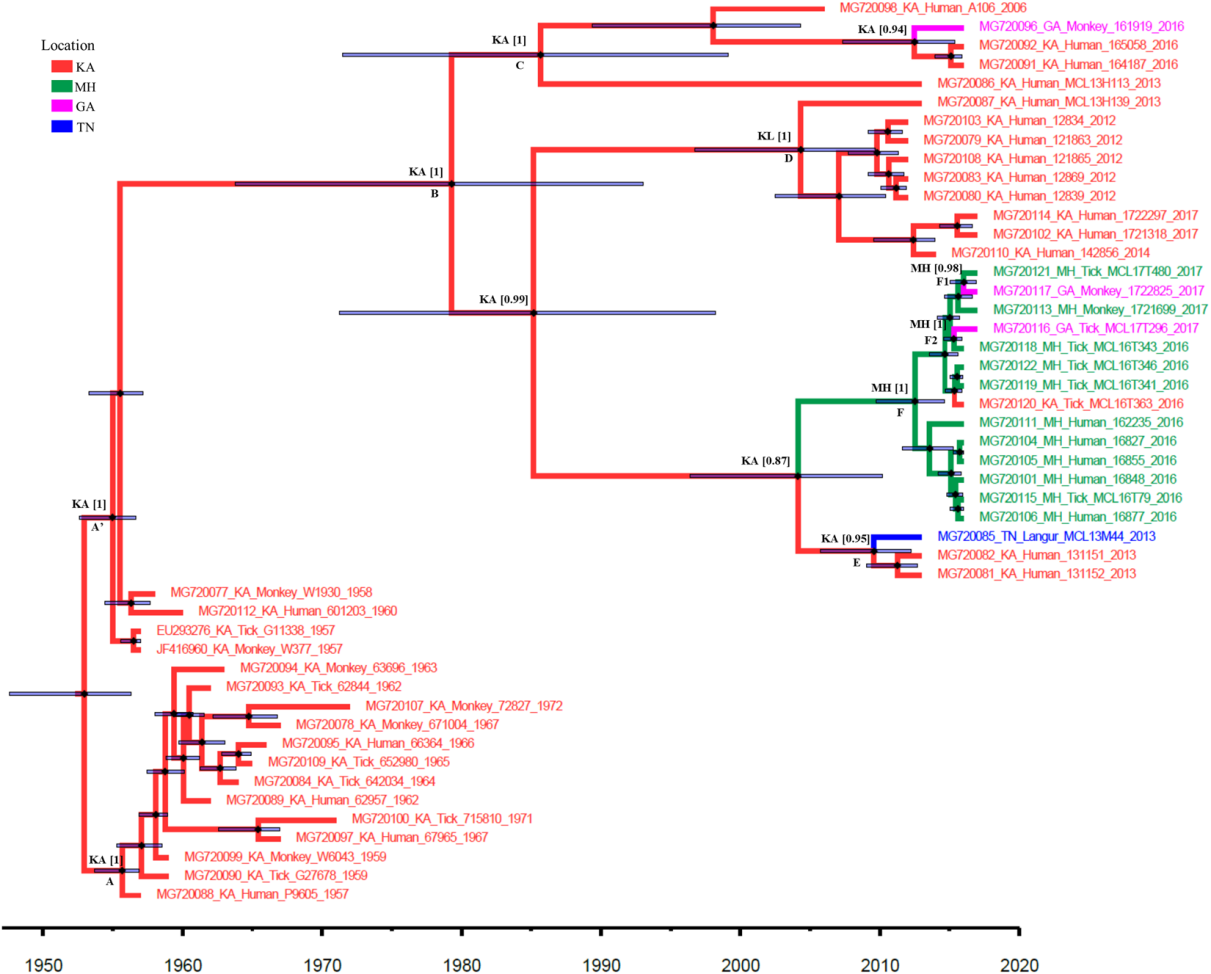
Maximum Clade Credibility (MCC) tree of KFDV whole gene: Maximum Clade Credibility (MCC) tree for KFDV based on whole genome sequences (n = 48): Key nodes are labeled. The circles at the nodes indicate the posterior clade probabilities with size reflecting the confidence. The 95% HPD limits of the tMRCA (Time to the Most Recent Common Ancestor) estimates are indicated as the translucent horizontal bars at the nodes. The numbers at the nodes correspond to the ancestral states with their probabilities. The branches are colored according to the respective ancestral geographical region. The geographical regions are represented by 2 letter codes (GA: Goa; KA: Karnataka; KL: Kerala; MH: Maharashtra; TN: Tamil Nadu).

The estimated mean rate of nucleotide substitution of KFDV was 4.2 × 10^−4^ subs/site/year with 95% Highest Posterior Density limits (HPD) (3.2, 5.35) x 10^−4^ based on the best fit uncorrelated exponential clock model (Table 4). Based on this rate, the inferred mean root age was 64 years (HPD: 60.7, 69.4 years), indicating an emergence time ~November-1952 (June- 1947, April-1956) (Fig. 2). The geographical ancestral state for KFDV was Karnataka with probability 1.0. Majority of the earliest strains (1959–1972) formed a cluster (*node A*) with the earliest reference strain P9605 of 1957 from a human host. The sequences of 1957 from tick and monkey and two other sequences of 1958 and 1960 were distinct and found to share a common ancestor with the more recent strains of 2006–2017 though with a low nodal posterior support of 0.44 (*node A’*). These recent strains had a divergence time estimate of 37.7 (24, 53) years back {March-1979 (1964–1993)} (*node B*) with Karnataka as the ancestral source (state probability 1). The A106 2006 isolate formed a sub-group with strains from Karnataka during the period 2013–2016 and a single monkey strain from Goa of 2016 (161919). The common ancestor of these strains (*node C*) emerged around 31 years ago (~1986) from Karnataka. An indigenous group of strains from Karnataka over the period 2012–2017 evolved with a common ancestor (*node D*) estimated ~2004. Two sub-groups emerging from nodes E and F were noted to have a common ancestor (*node G*) that emerged from Karnataka with lower state probability (0.88). An independent entry to Tamil Nadu is noted as strains of 2013 from Tamil Nadu and Karnataka emerged from a common ancestor ~July 2009 with Karnataka as the source (probability 0.94). Dispersion is observed in Maharashtra around June 2012 as the common ancestor of strains circulating over the period 2016–2017 emerged from *node F* with Maharashtra as the source (state probability 1). Two separate movements to Goa are also noted (*nodes F1 and F2*) in the form of isolates MCL17T296 and 1722825 having accession number MG720116 and MG720117 respectively.

**Table 4:** Estimates of substitution rates and root ages for KFDV sequences based on different clock models for the two datasets (a) Whole genomes (b) E-gene sequences.

Clock Model	Posterior	Marginal likelihood	Mean substitution rate x 10^−4^ (95% HPD) (subs per site year^−1^)	Root age (95% HPD) (years back)	Root age (95% HPD) (years)
a) Whole genomes					
Uncorrelated exponential	−25106.841 [−25134.062, −25079.784]	−22174.448 (±0.07)	4.2 (3.2-5.35)	64.0421 (60.70, 69.4)	November 1952 [June-1947, April-1956]
Uncorrelated lognormal	−25112.401 [−25136.159, −25088.453]	−22177.365 (±0.06)	3.9 (3.16-4.67)	63.5689 (61.07, 67.57)	June 1953 [May-1949, April-1955]
b) E-gene sequences					
Uncorrelated exponential	−6903.16 [−6945.708, −6861.445]	−3556.612 (±0.18)	5.4 (3.59-7.43)	64.82 (60.60-71.43)	February 1952 [June-1945, May-1956]
Uncorrelated lognormal	−6922.692 [−6959.007, −6886.008]	−3563.871 (±0.16)	4.6 (3.3-6.05)	64.88(61.14-70.2)	January 1952 [October-1946, October-1955]

#### Based on the E-gene dataset

**Figure 3:**
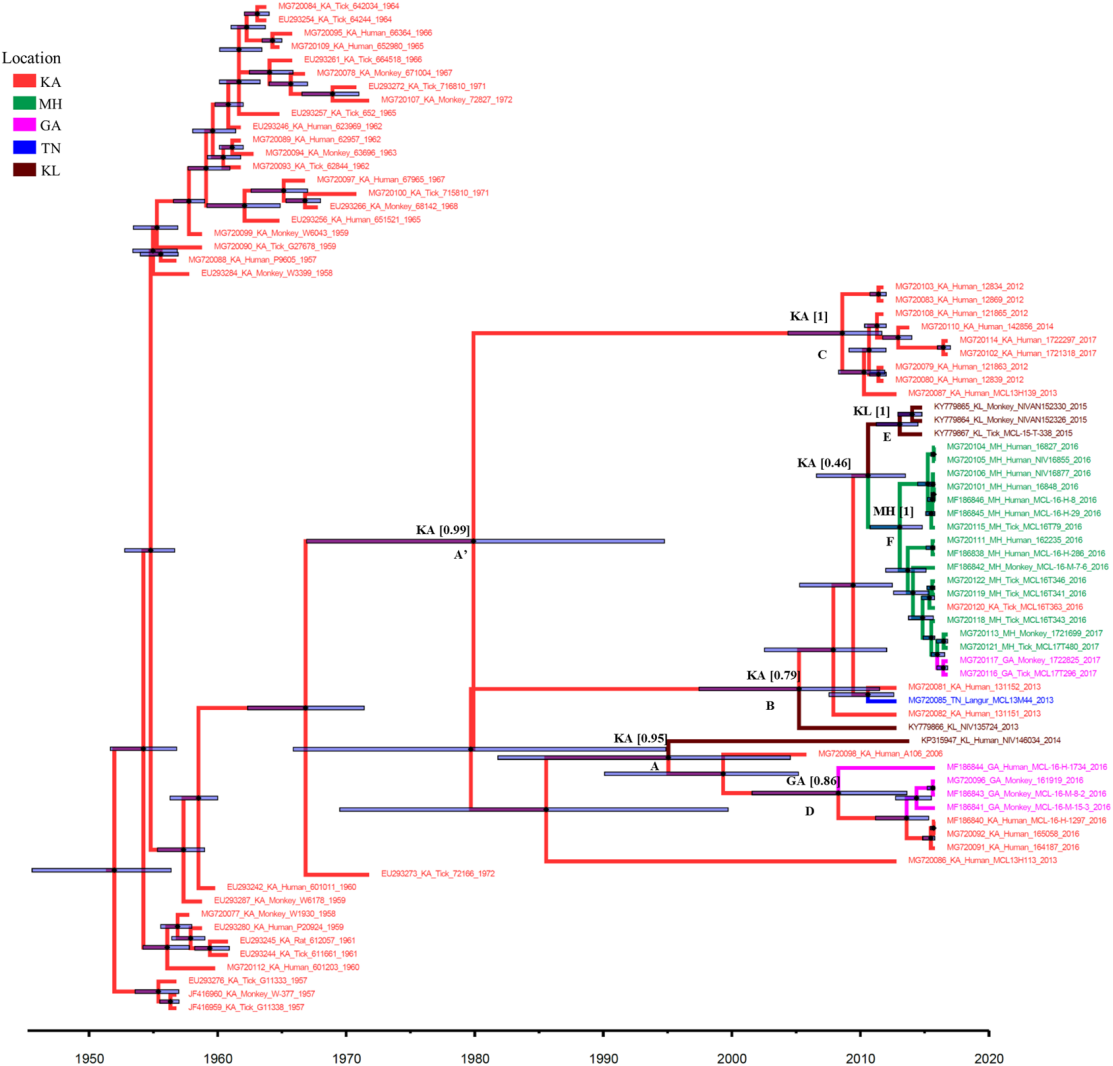
Maximum Clade Credibility (MCC) tree of KFDV E gene: Maximum Clade Credibility (MCC) tree for KFDV based on envelope gene sequences (n = 76) Key nodes are labeled. The circles at the nodes indicate the posterior clade probabilities with size reflecting the confidence. The 95% HPD limits of the tMRCA (Time to the Most Recent Common Ancestor) estimates are indicated as the translucent horizontal bars at the nodes. The numbers at the nodes correspond to the ancestral states with their probabilities. The branches are colored according to the respective ancestral geographical region. The geographical regions are represented by 2 letter codes (GA: Goa; KA: Karnataka; KL: Kerala; MH: Maharashtra; TN: Tamil Nadu).

The mean substitution rate for the E-gene was estimated to be 5.4 × 10^−4^ subs/site/year with 95% Highest Posterior Density limits (HPD) (3.59, 7.43) x 10^−4^ based on the uncorrelated exponential clock model (Table 4). The inferred root age was 64.8 years (HPD: 60.6, 71.4) implying its emergence around February-1952 (June-1945, May-1956). Figure 3, representing the MCC tree for the E-gene sequences, reveals three major sub-groups with strong posterior support (*nodes A, B*, and *C*) including viruses mainly of the period from 2012–2017, and one strain of 2006 from Karnataka (MG720098). These viruses shared a common ancestor (posterior support 0.97) with a strain (*Haemaphysalis kyasanurensis*) from Karnataka in 1972. Within the group emerging from *node A*, a sub-group of viruses from Karnataka and Goa, of 2016, diversified from a common ancestor ~ mid-2008 with Goa as the ancestral source with state probability 0.86 (*node D*). A strain from Kerala of 2014 (KP315947) falling in the group A was noted to have Karnataka as the source with state probability 0.95. Another Kerala isolate (KY779866) of 2013, was noted to have Karnataka as the ancestral source with probability 0.79 (node B). Within this group, a strain from Tamil Nadu (MG720085) of 2013 had Karnataka as the source with high state probability of 0.94, though the posterior support for this node was low (0.19). The tMRCA of this strain was estimated ~2011. Three Kerala isolates of 2015, having tMRCA ~2013 (*node E*), and several strains from Maharashtra with tMRCA ~2013 (*node F*), were also found to have Karnataka as the ancestral source though with a low probability 0.46. The two Goa strains of 2017 (MG720116 and MG720117) showed Maharashtra as the ancestral source with a high probability of 0.99. Further, the tMRCA of recent Karnataka viruses of the period 2012–2017 (*node C*) was estimated ~2009.

## Discussion

The KFDV is enzootic to India and maintained in *Haemaphysalis spinigera* ticks, as the main vector^23^, though at least 16 tick species^24^ and different species of mammals have been shown to be involved in the natural cycle of KFDV. Though molecular clock studies undertaken by earlier workers, gave the estimates of the rates of molecular evolution of the KFDV as well as the time scales of divergence including the time to the most recent common ancestor (tMRCA), limited information is provided regarding the viral dispersal patterns. Therefore, application of phylogeographic reconstruction was undertaken to study the dispersion pathways of KFD viruses within India. We undertook the complete genome sequencing of KFD viruses as well as additional 28 E-gene sequences as representative isolates from human, ticks and monkey samples collected from the KFD affected areas in the recent and past times. While selecting samples for the whole genome sequencing, it was ensured that there was no sampling bias. Thus though during the period, December 2011 - March 2012, the KFDV positivity was high (60%) in Karnataka, only 4 isolates were sequenced. The enlarged and more complete dataset thus generated was used for phylogeography and other evolutionary analyses. The study would help understand the evolution, spatiotemporal patterns of dispersal of the KFDV within Karnataka and between the affected states and so also determine estimates of the time of divergence of newly evolved sub-lineages of KFDV.

Based on the p-distance calculations, it was noted that the genomes of KFDV (1957–2017) are overall highly conserved (2.24%/0.75% divergence at *nt*/*aa* level). The level of divergence, especially in the 1957 to 1972 time frame, was found to be even lower (0.31%/0.30% at the *nt*/*aa* level). Though this considerably low genetic diversity is a surprising observation in case of vector borne *flaviviruses* in general, another tick-borne flavivirus, a KFDV variant, referred to as the Alkhurma hemorrhagic fever virus (AHFV), did show very low levels of diversity at the genomic level^8^ (1.1%/0.8% divergence at the *nt*/*aa* level) in isolates obtained from human cases over the course of 15 years. The low genetic diversity was also similar to that of an unrelated tick-borne member of the Bunyaviridae family, Rift Valley Fever Virus^25^. The lower diversity within KFDV isolates and specifically when the spread was restricted to the single state of Karnataka in the early time frame may thus reflect limited transmissibility of tick-borne viruses in comparison to mosquito-borne viruses.

The evolutionary rate obtained for KFDV based on whole genome as well as the E-gene sequences in this study was almost the same and is comparable to that obtained through the Bayesian coalescent analysis of partial E and NS5 gene sequences of around 50 KFDV (1957 to 2006) and AHFV^26,27^ isolates^21^. With a mean rate of 6.4 × 10^−4^ subs/site/year, the tMRCA for the KFDV isolates was estimated to have occurred ~1948, just nine years before the disease identification^21^. Another study^8^ based on a Bayesian coalescent phylogenetic analysis of few KFDV (3) and 18 AHFV full-length sequences, revealed a slower rate of evolution (9.2 × 10^−5^ subs/site/year). The present study which estimated the KFDV to have evolved around 1953 (based on whole genome data) or 1952 (based on E-gene data) therefore corroborates the findings based on partial KFDV sequences rather than the latter study including predominantly AHFV sequences and emphasizes that the evolutionary rates of tick-borne and mosquito-borne viruses are similar. The rate of substitution based on the E-gene is noted to be negligibly higher than that observed for the whole genomes and can be explained in terms of the higher percent nucleotide difference in the E-gene when compared to the whole genome (2.61% versus 2.24%).

Both the whole genome sequence data, as well as the E-gene sequences, revealed that all the earlier strains do not form a single homogenous cluster. Few strains of 1957–58 and 1960 have evolved distinctly and may be representing the spread within Shimoga district as well as other districts of Karnataka. Notably, the sequences of these strains (KA_601203, KA_W1930, KA_G11338 and KA_W-377) possessed a mutation A123T in the E gene as was noted in several of the later strains circulating in Maharashtra, Goa, Kerala and Tamil Nadu (Supplementary Tables S3 and S4). Significantly, the E-gene MCC tree (Fig. 3) reveals that a strain from 1972 (EU293273) of Karnataka (*Haemaphysalis kyasanurensis*) was distinct from the other strains and shared a common ancestor with the recent strains (2006–2017) with divergence time around 1967. Until 1971, it was known that KFDV was endemic to Shimoga district, but by 1972, a new focus of virus activity had appeared in Uttara Kannada district of Karnataka. This strain forming an outlier to the later KFDV strains from 2006 onwards, may have contributed to the spread of the virus outside Karnataka as well. Evolution of KFDV, in the recent past, involved diversification into at least four sub-lineages, based on high posterior support in the whole genome-based MCC tree. These sub-lineages corresponded majorly to evolution in different states, Karnataka, Goa, Maharashtra and Tamil Nadu. On the other hand, the E-gene tree (Fig. 3), revealed three sub-lineages with good support. No separate cluster could be identified for the Tamil Nadu strain of 2013 in this case.

The sub-lineage comprising sequences exclusively from the Karnataka state (2012–2017) was observed to have emerged around 2004 for the whole genome (*node D*) and 2009 for E-gene (*node C*). This covers the time span when during 2011–2012, in-spite of vaccination programs; increased KFDV positivity was noted in Karnataka^15,16^. In November 2012, the death of monkeys (*Macaca radiata* and *Presbytis entellus*) was reported from the Bandipur National Park, in Chamarajanagar another district of Karnataka and typical KFD-like clinical symptoms were noticed from humans who were involved in handling and incineration of the sick monkeys. The indigenous evolution of KFDV as noted in this group of strains, involved the diversification to involve several *aa* substitutions, namely in prM: G85S (except in MCL13H139), A133V; E: G479A; NS1: A176T, A247V, V278I; NS2: V24I, F111L (in strains of 2012), R150K; NS3: V253A; NS4: S78N and S222N (both in the strains of 2012); and NS5: E527K, K862R. The possible causes for the upsurge and spread could, therefore, be the inadequate or limited efficacy of vaccination as well as the occurrence of possibly significant mutations in the virus strains.

In addition, a spillover of KFD cases to the border areas in the neighboring states of Tamil Nadu, Goa, Maharashtra, and Kerala^28^ has also been noted. Dispersal of the viruses to similar eco-regions in the bordering states may be accomplished through the movement of animals presumably carrying ticks. The movement of KFDV from Karnataka to Goa around mid-2008 is indicated from the analysis of the E-gene sequence data (Fig. 3). Further, an exportation of the virus from Goa to Karnataka was also noted in the form of isolates MG720092, MG720091 of 2016 from Belgaum, Karnataka. This can be explained as cases of KFDV in cashew nut workers contracted in Goa during cashew nut harvesting from a KFD-affected village in Sattari taluka of Goa^18^. Based on the whole-genome data, some of the strains within this sub-lineage were found to possess unique substitutions in certain genomic regions: prM: 4RL, V152A; NS2: V47A, F120I, M160L, T244A; NS3: Q216R, K585R; NS4: A328T, M388I; and NS5: K101R, A279S, D787G. No specific mutation relating to this group was found in the E-gene.

The emergence time of the KFDV transmissions to Maharashtra are noted from Karnataka with tMRCA ~mid-2012 (whole-genome) and ~2013 (E-gene). Within this group, two sub-groups could be delineated, one consisting of only the sequences from Maharashtra of 2016 and the other consisting of sequences from Maharashtra, Goa, and Karnataka of the period 2016–17. Recent multiple virus transmissions from Maharashtra to Goa were noted in the form of the Goa isolates of 2017. Together, these findings indicate that the State of Maharashtra represents a new source for transmission of KFDV. The group possesses a unique aa substitution NS4: I107T. Based on whole genome sequences, an independent dispersion to Tamil Nadu is also noted from Karnataka in the form of a separate sub-group (MG720085). This sub-group of strains having tMRCA approximately around mid-2009 is noted to have unique aa substitutions in prM: K67R, NS4: T234A; NS5: R244K. No mutation specific to these strains in the E-gene, explains the reason why no separate group could be identified in the E-gene based phylogeny. Notably, all the strains corresponding to the Maharashtra and Tamil Nadu sub-lineage (node G, in Fig. 2) possessed common mutations, C: I106F, A109V, T113A, F116L; E: D239N; NS1: V139I; NS2: M40V, V167A; NS5: Y133H, A156V, T564A, V724A, G826E. The mutation D239N in the E-gene was also noted in the additional strains from Kerala of 2013–2015 (Node B, in Fig. 3).

**Figure 4:**
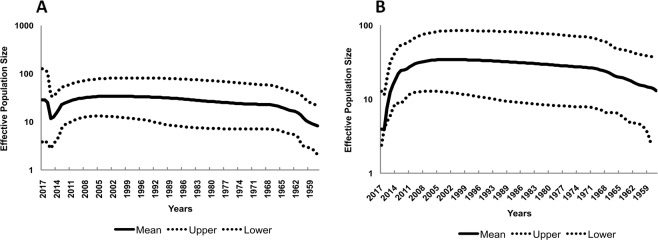
Bayesian skyline plot: Bayesian skyline plot for KFDV isolates based on (**a**) whole genome sequences (**b**) E-gene sequences.

**Figure 5:**
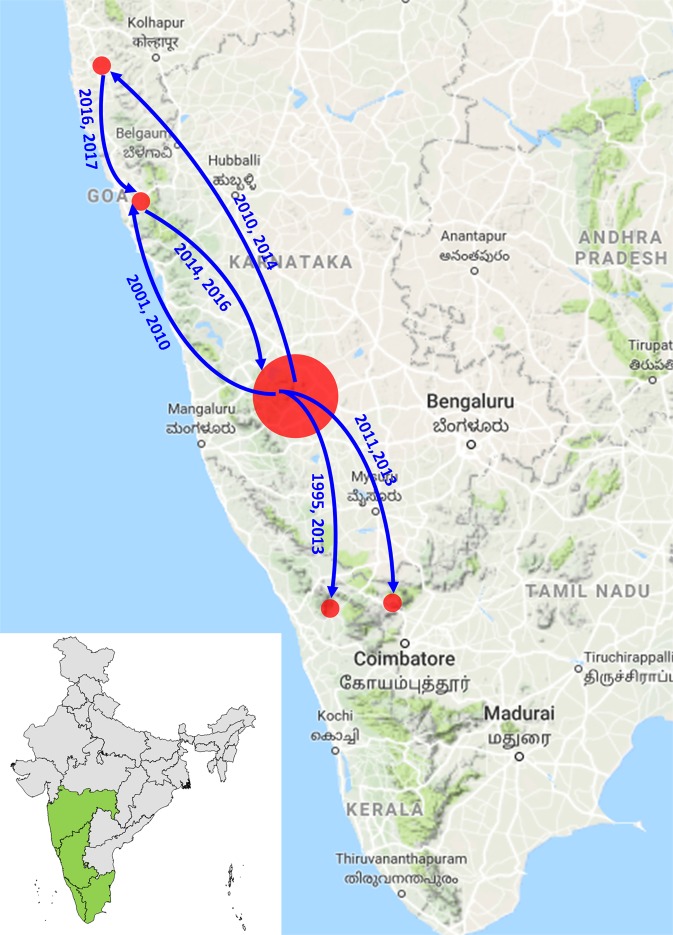
Migratory pathways and migration times of KFDV: Schematic representation of the plausible migratory pathways and migration times of KFDV on a map of the study area. Numbers along the arrows indicate the year of migration and introduction into the geographic locations representing the specific Indian State. Map not to scale.

To study the migration patterns of the KFDV, the transition rates with significant BF values (>3) and posterior probability values (>0.8) were identified (Supplementary Table S5). Significant BF values and posterior probability values were noted between Karnataka/Goa, Goa/Maharashtra, Karnataka/Tamil Nadu, Karnataka/Maharashtra and Maharashtra/Tamil Nadu. The Bayesian skyline plot (Fig. 4A,B) reflecting the genetic diversity of KFDV reveals an almost stable phase or slightly increasing trend over the period from 1955 to 2015, followed by an increase beyond this period which is more remarkable from the whole genome data (Fig. 4B). This trend is reflective of the proliferation of the virus to the neighboring states of Karnataka and also the recent exchange of viruses between neighboring states. Figure 5 provides a schematic representation of the migratory pathways along with the migration times, on a map of the study area. As is clear from this figure, the transmissions of KFDV are along the narrow mountainous stretch, known as the Sahyadri range or Western Ghats, parallel to the western coast of the Indian peninsula traversing the States of Kerala, Tamil Nadu, Karnataka, Goa, and Maharashtra. The free movements of tick-infested monkeys in these forests along with changes in agricultural and occupational practices that encourage close proximity to humans and/or their dwellings can explain the KFDV spread from the Karnataka epicenter to movements between the states.

Mapping of the sites noted to showing evidence of positive selection, as well as major mutational sites on the three-dimensional E-protein structure models, was undertaken for evaluating possible functional correlations. Though among the five sites predicted to be positively selected, the sites prM: 152 V/I/A, E: 123 A/T, NS2: 357 G/S/C and NS3: 14 R/G/T (1505) could be mapped to specific functional domains of these proteins, further, studies may be warranted for experimental examination of their functional role. Similarly the role of other substitutions noted in the E-protein such as D239N, G230E would need further investigation. Notably certain specific mutational changes (in the C, prM, NS1 and NS3 proteins) were noted between the earlier strains of the period 1957–1972 and the recent strains (2006–2018) wherein the virus was noted to have spread its geographical distribution from Karnataka to other states Maharashtra, Goa, Tamil Nadu, and Kerala stretching from north to south in the Western Ghats. The fact that an indigenous group of strains from Karnataka also possessed these mutations, indicate that the mutations are not a factor for viral adaptation to the terrain. The mutations could have facilitated the transmission to the newer areas through some advantage to the virus in the vector or hosts, though it cannot be denied that they could be the accumulation of random mutations. Unfortunately, the non-availability of KFDV isolates during the interim period due to lack of a well-equipped containment laboratory then and the absence of experimental evidence at this time point does not permit a more definite interpretation. Similarly though mutations have been noted in several genes specific to the different phylogenetic groups reported in this study and corresponding to different geographical locations, experiments are necessary to identify any possible significance of these mutations.

In order to understand whether the passage history could influence the introduction of mutations in the sequenced isolates, we compared the whole genome sequences of representative isolates at different passage levels (data not shown). It was found that tissue culture passage could lead to negligibly low nucleotide substitutions (upto to three) and upto two amino acid mutations at passage levels three and above. None of the mutational sites showed evidence of being positively selected (data not shown) and most of the isolates in this study are of passage 1 and 2, indicating the robustness of the findings of this study.

In summary, the present genomic and phylogeography study revealed the evolution of the virus and also the regions linked with the transmission dynamics of the KFDV. The earliest migratory routes to Kerala, Maharashtra and Tamil Nadu, as well as Goa, are most likely from the State of Karnataka. Thereafter exchange of viruses between neighboring regions is being noted in the recent past. The study highlights that the ongoing transmissions need to be curtailed through intense surveillance activities and enhanced vaccination coverage efforts.

## Materials and Methods

### Ethics statement

This study was prior approved by the scientific advisory committee, Institutional Animal Ethics committee (IAEC) and adhered the regulation of Committee for the Purpose of Control And Supervision of Experiments on Animals (CPCSEA). The lyophilized old KFD virus isolates (mice brain suspension) or propagated in *BHK-21* tissue culture were used for sequencing the genome. The virus isolation was attempted from KFD positive blood/serum samples in *BHK-21* cells. The standard international guidelines and Institutional Human Ethics committee (IHEC) guidelines were followed to anonymized the identity of KFD cases. Prior approval was obtained from IHEC NIV/IEC/2018/D-9 for including these isolates in the study. The informed consent was taken for recent human samples.

### KFD virus isolation (*in vitro* and *in vivo* method)

Old KFDV (lyophilized virus mouse brain suspensions) isolated during the year 1957–1972 were procured from the virus repository of ICMR-National Institute of Virology, Pune, India. The isolates were stored in cold chain and were used directly in the study after reconstituting in 10% phosphate buffer supplemented with bovine serum (BAPS). The recent KFDV positive clinical specimens spanning the year 2006 to 2018 were used for virus isolation, in CD8 infant mouse and BHK-21 cells, by selecting samples from different geographical locations, hosts, and vectors. These samples [monkey (9), human (25), and tick pools (13)] were referred/collected during different KFD outbreaks.

The samples having Ct value less than 28 were selected for genome sequencing. In the absence of the older clinical samples and to ensure adequate material for primer sequencing using the traditional Sanger sequencing protocol, virus isolation for the recent clinical samples also was undertaken. For *in vivo* propagation, serum samples/tick homogenates/monkey tissue homogenates were inoculated in infant mice and observed for sickness on daily basis. On sickness, the mice brain was extracted, homogenate and suspended in 10% BAPS and stored at −80 °C until further use. The KFDV were propagated in *BHK-21* cells at 37 °C for 5 days, and the supernatants were harvested for RNA extraction when cytopathic effects (CPE) were observed. The tissue culture fluid (TCF) containing viral particles were obtained from cell lysates after three freeze-thaw cycles. The cells were incubated with either mice brain suspension or TCF was used for the total RNA extraction and for sequencing of KFDV genomes. The virus propagation experiments were performed in biosafety level 4 (BSL-4) laboratories and inactivated. After inactivation of the virus in trizol reagent (Invitrogen), the vials were moved in a BSL-2 facility with proper precautions and safety measurements for RNA extraction and sequencing.

### KFD virus sequencing using *Sanger’s method*

The KFDV isolates were aliquoted and inactivated in the BSL-4 facility. RNA was extracted from KFDV isolates in BSL-2 facility, by using Trizol Reagent (Invitrogen), followed by the QIAamp Viral RNA Mini Kit (Qiagen) column purification. The extracted RNA was stored at −80 °C until use. A set of 50 primers were designed, using the reference KFDV strain P9605 (Accession number: JF416958), for the whole genome sequencing of KFDV that are enlisted in Supplementary Table S5. The primer combinations used for the RT-PCR are given as Supplementary Table S6. The cDNA synthesis undertaken by using Invitrogen Superscript III Taq Polymerase enzyme; with the reaction conditions as 50 °C (30 m), 94 °C (2 m), 94 °C (15 s), 50 °C (1 m), and 68 °C (1 m 30 s). Final extension was performed at 68 °C (10 m) for 40 cycles. The primer pairs 9 F + 11 R, 17 F + 19 R, and 24 F + 26 R did not require the additional 30 sec, during the extension period.

The cDNA products from the above step were electrophoresed on 1.5% Agarose gel. The products were excised and purified through the QIAquick Gel Extraction Kit (Qiagen). The purified cDNA products were subjected to the cyclic Sequencing PCR, by using BigDye™ Terminator Cycle Sequencing Kit (Invitrogen). The cyclic sequencing reaction conditions followed were 96 °C (1 m), 50 °C for (5 s), and 60 °C (4 m), for 25 cycles. Further, the products were purified by using DyeEx 2.0 kit (Qiagen) and sequencing was performed using the ABI PRISM® 3100 Automated DNA Sequencer. The results of the sequenced chromatogram data were assembled by using the Sequencher 5.1 software (Accelrys Inc.).

### KFD virus sequencing using *next-generation sequencing method*

One mL of mice brain suspension/tissue culture fluid was used for RNA extraction. RNA library preparation and its quantification were performed using the protocol described by Yadav *et al*.^29^, and loaded in the Illumina Miniseq platform. The FASTQ files generated after the completion of the run were analyzed using CLC Genomics Workbench software version 10.1 (CLC, Qiagen).

The list of 46 KFDV isolates of this study from different sources (tick pool, monkeys, and human) along with their sampling years, locations, passage history and their GenBank accession numbers are provided (Supplementary Table S1). Of a total of 46 isolates, Sanger sequencing was done for 40 isolates while six isolates were sequenced using the next-generation sequencing method. Additionally five isolates were sequenced by both the methods to check for concurrency.

### Phylogeny and genetic diversity

The KFDV whole genome sequences obtained in this study (n = 46) and a two whole genome available of the KFDV reference strain from India (G11338 and W-377) isolated in 1957 (GenBank accession number JF416959.1 and JF416960.1) were aligned using MEGA and the length of the alignment was restricted to 10,248nt. Phylogeny was carried out using the neighbor-joining method available in MEGA v6^30^ using the Kimura 2-parameter nucleotide (*nt*) substitution model with 1000 bootstrap replicates. The proportion of nt differences was estimated by pairwise comparisons using the p-distance model. Estimation of the gene-specific ratio of nonsynonymous to synonymous substitutions (dN/dS) was also done in MEGA.

### Selection pressure and functional analyses

Selection pressure analysis based on the whole genome dataset and also on the enlarged dataset of the E-gene region was performed in the Datamonkey server^31^ using the single likelihood ancestor counting (SLAC), fixed effects likelihood (FEL), random effects likelihood (REL), mixed effects model of evolution (MEME) and (Fast, Unconstrained Bayesian AppRoximation) FUBAR methods. Codon sites were identified to be under positive selection pressure based on the statistical significance level (P ≤ 0.1 for SLAC, FEL, and MEME, Bayes factor >50 for REL or posterior probability >0.9 for FUBAR) by at least two of the methods. To co-relate, the mutational sites to functional sites, the available protein structural data of other flaviviruses was used. The E-protein of KFDV was modeled as a trimer using the crystal structure of TBEV (506 A.PDB) as a template the sequence of which showed 80% identity with the KFDV sequence (strain P9605). The SWISS-MODEL server (http://swissmodel.expasy.org/interactive#structure)^32^ was used for homology modeling and validation of the modeled structure was carried out using the PROCHECK tool from structure analysis and verification server (SAVES) [https://services.mbi.ucla.edu/SAVES/]. The 3D structure of the protein was visualized using BIOVIA Discovery Studio Visualizer (www.accelrys.com/products).

### Phylogeographic reconstruction of KFDV and migration pattern

The phylogeographic analyses based on 48 KFDV whole genome sequences was carried out in this study. Representative isolates based on sampling time and geographic location were selected for the study to ensure that there was no sampling bias. In addition, the analysis was also carried out on a separate dataset of 76 E-gene sequences by including the E-gene data that was available in GenBank (Supplementary Table S8). The best-fit model of nucleotide substitution selected on the basis of Akaike Information Criterion (AIC) in jmodeltest-2.x was found to be the GTR (General Time Reversible) + G4 (gamma with 4 categories) + I (proportion of invariant sites) model for the for either dataset. Temporal information (year of isolation) of sequence data was used to estimate the evolutionary rate and ancestral times, by generating a maximum clade credibility (MCC) tree using the Bayesian Markov chain Monte Carlo (MCMC) approach as implemented in BEAST 1.8.2^33^ employing the relaxed uncorrelated lognormal and uncorrelated exponential clock models with the Bayesian Skyline tree prior. Two independent runs of the chain were carried out, each with 50 million generations and sampling frequency of 1000 and combined with 20% burn-in for the whole genome and the E-gene datasets. The convergence of the chain was analyzed by using Tracer1.5 and effective sample size (ESS) values of >200 indicated sufficient level of sampling. The MCC tree was visualized using FigTree 1.4.2. The spatial information (state of isolation) of the isolates was further used to infer the geographical dispersal patterns of the virus by fitting a standard symmetric substitution with the Bayesian stochastic search variable selection (BSSVS)^34^ in BEAST. Four geographical locations (Karnataka, KA; Tamil Nadu, TN; Maharashtra, MH; Goa, GA) were selected and coded as discrete states. The MCC tree obtained was input to the program SPREAD 1.0.5^35^ to visualize and analyze the transmission pathways. To infer diffusion rates between any pair of locations Bayes Factor (BF) values available in SPREAD, were calculated.

## Supplementary information

Supp Table 1,2,5-8.

Supp Table 3.

Supp Table 4.

## Data Availability

The datasets analyzed during the current study are available in the Genbank database.
