# Supplementary material for: Phylogeography of Kyasanur Forest Disease virus in India (1957–2017) reveals evolution and spread in the Western Ghats region
Source: Sci Rep. 2020 Feb 6;10:1966. doi: 10.1038/s41598-020-58242-w (PMC7005018; doi:10.1038/s41598-020-58242-w)
Supplement: Supplementary file 1 — Supp Table 1,2,5-8. [file 41598_2020_58242_MOESM1_ESM.doc]

**Phylogeography of Kyasanur Forest Disease virus in India (1957- 2017) reveals evolution and spread in the Western Ghats region**

Pragya D Yadav^1^, Savita Patil^1^, Santoshkumar M. Jadhav^2^, Dimpal A. Nyayanit^1^, Vimal Kumar^1^, Shilpi Jain^1^, Jagadish Sampath^1^, Devendra T. Mourya^1^, Sarah S. Cherian^2*^

^1^Maximum Containment Laboratory, ICMR-National Institute of Virology, Sus Road, Pashan, Pune 411021, India

^2^Bioinformatics Group, ICMR-National Institute of Virology, Pune 411001, India

**Supplementary Files:**

1. **Supplementary Table S1:** Details of 46 whole genomes of the KFDV isolates generated in this study.
2. **Supplementary Table S2** The RNA concentration, the total number of reads number of reads and the mapped reads obtained from KFDV RNA samples.
3. **Supplementary Table S3** (Excel sheet): Amino acid substitutions in the different genes of KFDV based on 46 whole genomes.
4. **Supplementary Table S4** (Excel sheet): Amino acid substitutions observed in the E-gene sequences (n=76)
5. **Supplementary Table S5:** Bayes Factor and posterior probability values supporting the migration links between the different geographical regions for KFDV based on the whole genomes and E-gene datasets. Links supported by statistically significant migration links (BF value >3) are shown in bold.
6. **Supplementary Table S6**: List of Primers used for whole genome sequencing of KFDV using Sanger’s method.
7. **Supplementary Table S7:** Primer Combinations and Positions in the reference KFDV genome.
8. **Supplementary Table S8:** Details of additional 28 E-genes from KFDV isolates obtained from GenBank.

**Supplementary Table S1: Details of the 46 whole genomes of the KFDV isolates generated in this study.**

| **S. No.** | **ID No.** | **Year** | **Location** | **Ct Value of clinical samples** | **Isolation Source (host)** | **Sequencing method** | **Passage #** | **Sample type** | **Accession No.** |
| --- | --- | --- | --- | --- | --- | --- | --- | --- | --- |
| 1 | NIV_W1930 | 1958 | India: Chimnoor, Karnataka | NA | *Semnopithecus entellus* | Sanger | P-4 | M.Br. | MG720077 |
| 2 | NIV_671004 | 1967 | India: Dharwad, Karnataka | NA | *Semnopithecus entellus* | Sanger | P-1 | M.Br. | MG720078 |
| 3 | NIV_121863 | 2012 | India: Shimoga, Karnataka | 22.3 | *Homo Sapiens* | Sanger | P-2 | M.Br. | MG720079 |
| 4 | NIV_12839 | 2012 | India: Shimoga, Karnataka | 22 | *Homo Sapiens* | NGS* | P-6 | *BHK-21*, TCF | MG720080 |
| 5 | NIV_131152 | 2013 | India: Shimoga, Karnataka | 27.5 | *Homo Sapiens* | NGS | P-3 | M.Br. | MG720081 |
| 6 | NIV_131151 | 2013 | India: Shimoga, Karnataka | 26 | *Homo Sapiens* | NGS | P-3 | M.Br. | MG720082 |
| 7 | NIV_12869 | 2012 | India: Shimoga, Karnataka | 21.6 | *Homo Sapiens* | Sanger | P-2 | M.Br. | MG720083 |
| 8 | NIV_642034 | 1964 | India: Shimoga, Karnataka | NA | *Haemaphysalis turturis* | Sanger | P-5 | M.Br. | MG720084 |
| 9 | NIV_AN131058 | 2013 | India: Nilgiri, TamilNadu | 26.8 | *Presbytes Entellus* (Langur) | NGS | P-5 | M.Br. | MG720085 |
| 10 | NIV_137459 | 2013 | India: Shimoga, Karnataka | 26.91 | Homo Sapiens | NGS | P-1 | M.Br. | MG720086 |
| 11 | NIV_134787 | 2013 | India: Uttar kannada,Karnataka | 22.4 | *Homo Sapiens* | NGS | P-5 | M.Br. | MG720087 |
| 12 | NIV_p9605 | 1957 | India: Shimoga, Karnataka | NA | *Homo Sapiens* | NGS | P-5 | M.Br. | MG720088 |
| 13 | NIV_62957 | 1962 | India: Gadag, Karnataka | NA | *Homo Sapiens* | Sanger | P-1 | *Vero*, TCF | MG720089 |
| 14 | NIV_G27678 | 1959 | India: Kopalgadde, Karnataka | NA | *Haemaphysalis*  *spinigera* | Sanger | P-3 | *Vero* | MG720090 |
| 15 | NIV_164187(1297) | 2016 | India: Belgaum, Karnataka | 25 | *Homo Sapiens* | Sanger | P-1 | M.Br. | MG720091 |
| 16 | NIV_165058(1644) | 2016 | India: Belgaum, Karnataka | 31 | *Homo Sapiens* | Sanger | P-1 | M.Br. | MG720092 |
| 17 | NIV_62844 | 1962 | India: Gadag, Karnataka | NA | *Haemaphysalis spinigera* | Sanger | P-1 | *Vero*, TCF | MG720093 |
| 18 | NIV_63696 | 1963 | India: Shimoga, Karnataka | NA | *Semnopithecus entellus* | Sanger | P-1 | *Vero*, TCF | MG720094 |
| 19 | NIV_66364 | 1966 | India: Sagar, Karnataka | NA | *Homo Sapiens* | Sanger | P-2 | M.Br. | MG720095 |
| 20 | NIVAN161919(m8/2) | 2016 | India: Caranzalem, Goa | 23 | *Semnopithecus entellus* | Sanger | P-1 | M.Br. | MG720096 |
| 21 | NIV_67965 | 1967 | India: Sagar, Karnataka | NA | *Homo Sapiens* | Sanger | P-1 | *Vero*, TCF | MG720097 |
| 22 | NIV_A106 | 2006 | India: Chikballapura, Karnataka | NA | *Homo Sapiens* | Sanger | P-2 | M.Br. | MG720098 |
| 23 | NIV_W6043 | 1959 | India: Belisiri, Karnataka | NA | *Semnopithecus entellus* | Sanger | P-3 | M.Br. | MG720099 |
| 24 | NIV_71580 | 1971 | India: Gunjnur, Karnataka | NA | *Haemaphysalis spinigera* | Sanger | P-3 | M.Br. | MG720100 |
| 25 | NIV_16848 | 2016 | India: Sindhudurg, Maharashtra | 22 | *Homo Sapiens* | Sanger | P-2 | M.Br. | MG720101 |
| 26 | NIV_1721318 | 2017 | India: Shimoga, Karnataka | 29 | *Homo Sapiens* | Sanger | P-3 | *BHK-21*, TCF | MG720102 |
| 27 | NIV_12834 | 2012 | India: Shimoga, Karnataka | 24 | *Homo Sapiens* | Sanger | P-2 | M.Br. | MG720103 |
| 28 | NIV_16827 | 2016 | India: Sindhudurg, Maharashtra | 26.26 | Homo Sapiens | Sanger | P-1 | M.Br. | MG720104 |
| 29 | NIV_16855 | 2016 | India: Sindhudurg, Maharashtra | 26 | *Homo Sapiens* | Sanger* | P-3 | *BHK-21*, TCF | MG720105 |
| 30 | NIV_16877 | 2016 | India: Sindhudurg, Maharashtra | 22 | *Homo Sapiens* | Sanger* | P-3 | *BHK-21*, TCF | MG720106 |
| 31 | NIV_72827 | 1972 | India: Holekoppa, Karnataka | NA | *Semnopithecus entellus* | Sanger | P-3 | M.Br. | MG720107 |
| 32 | NIV_121865 | 2012 | India: Shimoga, Karnataka | 24.3 | *Homo Sapiens* | Sanger | P-2 | M.Br. | MG720108 |
| 33 | NIV_652980 | 1965 | India: Shimoga, Karnataka | NA | *Haemaphysalis* | Sanger | P-1 | M.Br. | MG720109 |
| 34 | NIV_142856 | 2014 | India: Shimoga, Karnataka | 25 | *Homo Sapiens* | Sanger | P-2 | M.Br. | MG720110 |
| 35 | NIV_162235 | 2016 | India: Sindhudurg, Maharashtra | 26 | *Homo Sapiens* | Sanger | P-3 | *BHK-21*, TCF | MG720111 |
| 36 | NIV_601203 | 1960 | India: Tudikoppa, Karnataka | NA | *Homo Sapiens* | Sanger | P-1 | M.Br. | MG720112 |
| 37 | NIV_1721699 | 2017 | India: Sindhudurg, Maharashtra | 15 | *Semnopithecus entellus* | Sanger* | P-3 | *BHK-21*, TCF | MG720113 |
| 38 | NIV_1722297 | 2017 | India: Shimoga, Karnataka | 19 | *Homo Sapiens* | Sanger | P-3 | *BHK-21*, TCF | MG720114 |
| 39 | MCL_16_T_79 | 2016 | India: Sindhudurg, Maharashtra | 25.31 | *Ambliyomma* | Sanger | P-2 | M.Br. | MG720115 |
| 40 | MCL_17_T_296 | 2017 | India: North Goa, Goa | 22 | *Haemaphysalis spinigera* | Sanger* | P-3 | *BHK-21*, TCF | MG720116 |
| 41 | NIVAN1722825 | 2017 | India: North Goa, Goa | 18 | Black faced grey langur/Adult Male | Sanger | P-3 | *BHK-21*, TCF | MG720117 |
| 42 | MCL_16_T_343 | 2016 | India: Sindhudurg, Maharashtra | 20 | *Haemaphysalis* | Sanger | P-2 | *BHK-21*, TCF | MG720118 |
| 43 | MCL_16_T_341 | 2016 | India: Sindhudurg, Maharashtra | 22 | *Haemaphysalis* | Sanger | P-2 | *BHK-21*, TCF | MG720119 |
| 44 | MCL_16_T_363 | 2016 | India: Shimoga, Karnataka | 24 | *Haemaphysalis* | Sanger | P-2 | *BHK-21*, TCF | MG720120 |
| 45 | MCL_17_T_480 | 2017 | India: Sindhudurg, Maharashtra | 18 | *Haemaphysalis spinigera* | Sanger | P-3 | *BHK-21*, TCF | MG720121 |
| 46 | MCL_16_T_346 | 2016 | India: Sindhudurg, Maharashtra | 20 | *Haemaphysalis* | Sanger | P-2 | *BHK-21*, TCF | MG720122 |

* Both Sanger and NGS methods of sequencing performed for these samples

**Supplementary Table S2:** The RNA concentration, the total number of reads number of reads and the mapped reads obtained from KFDV RNA samples.

| **S. NO.** | **IDENTITY** | **QUBIT RNA CONC. (NG/µL)** | **KAPA QUANTIFICATION (NM)** | **TOTAL READS** | **MATCHED READS** | **PERCENT OF READS USEFUL** |
| --- | --- | --- | --- | --- | --- | --- |
| 1 | MG720081 | 12100 | 67 | 12,52,004 | 50,321 | 4 |
| 2 | MG720082 | 15500 | 71 | 7,01,838 | 39,425 | 5.6 |
| 3 | MG720086 | 648 | 224 | 29,21,002 | 5,45,605 | 18.7 |
| 4 | MG720087 | 105 | 214 | 28,08,894 | 3,15,834 | 11.2 |
| 5 | MG720085 | 460 | 450 | 20,10,484 | 4,74,112 | 23.6 |
| 6 | MG720088 | 412 | 385 | 20,78,682 | 3,15,664 | 15.2 |
| 7 | MG720080 | 510 | 269 | 24,67,426 | 2,21,385 | 8.97 |

**Supplementary Table S5:** Bayes Factor and posterior probability values supporting the migration links between the different geographical regions for KFDV for the whole genomes and E-gene datasets. Links supported by statistically significant migration links (BF value >3/ Posterior probability >0.8) are shown in bold.

| **Whole genomes** | | | | **E-genes** | | | |
| --- | --- | --- | --- | --- | --- | --- | --- |
| **FROM** | **TO** | **BAYES FACTOR** | **POSTERIOR PROBABILITY** | **FROM** | **TO** | **BAYES FACTOR** | **POSTERIOR PROBABILITY** |
| **KA** | **MH** | **62.76** | **0.98** | **KA** | **MH** | **3.37** | **0.84** |
| KA | GA | 0.34 | 0.23 | **KA** | **GA** | **7138.19** | **1.00** |
| **KA** | **TN** | **83.89** | **0.99** | KA | TN | 0.39 | 0.38 |
| KA | KL | 0.31 | 0.21 | **MH** | **GA** | **249.23** | **1.00** |
| **MH** | **GA** | **30.84** | **0.96** | **MH** | **TN** | **9.90** | **0.94** |
| **MH** | **TN** | **88.79** | **0.99** | GA | TN | 0.41 | 0.40 |
| MH | KL | 2.03 | 0.64 |  |  |  |  |
| GA | TN | 1.00 | 0.47 |  |  |  |  |
| GA | KL | 1.02 | 0.47 |  |  |  |  |
| TN | KL | 0.38 | 0.25 |  |  |  |  |

**Supplementary Table S6: List of Primers used for whole genome sequencing of KFDV using Sanger’s method.**

| **S. No.** | **Primers** | **Primer Sequence** |
| --- | --- | --- |
| 1 | KFD 1F (52-66) | ATG GCC AAA GGA GCC GTC |
| 2 | KFD 2F (170-290) | CAA GCG ATT CTG GGC GAC |
| 3 | KFD 2R (245-228) | GTC GCC CAG AAT CGC TTG |
| 4 | KFD 3F (529-591) | GAT GAT TCA ATC ATG TAC GAG TG |
| 5 | KFD 3R (551-530) | CAC TCG TAC ATG ATT GAA TCA TG |
| 6 | KFD 4F (790-810) | CCG ACA AGG TTC ATT GTC ATA |
| 7 | KFD 4R (861-841) | TAT GAC AAT GAA CCT TGT CGG |
| 8 | KFD 5F (1200-1300) | CAT TGT GGC TTG TGC CAA G |
| 9 | KFD 5R (1248-1230) | CTT GGC ACA AGC CAC AAT G |
| 10 | KFD 6F (1600-1700) | GAA CCG CAY GCT GTG AAA ATG |
| 11 | KFD 6R (1730-1710) | CAT TTT CAC AGC RTG CGG TTC |
| 12 | KFD 7F (2000-2100) | CAT CTA TGT TGG TGA GCT GAG |
| 13 | KFD 7R (2060-2040) | CTC AGC TCA CCA ACA TAG ATG |
| 14 | KFD 8F (2600-2700) | GTG GAG GCT GTG CTC AAC |
| 15 | KFD 8R (2610-2593) | GTT GAG CAC AGC CTC CAC |
| 16 | KFD 9F (3100-3200) | CAA TCA CAT TCC TGG GTA YGC |
| 17 | KFD 9R (3170-3150) | GCR TAC CCA GGA ATG TGA TTG |
| 18 | KFD 10F (3660-3760) | GTT TGA CAT GAG GAC YGG C |
| 19 | KFD 10R (3711-3693) | GCC RGT CCT CAT GTC AAA C |
| 20 | KFD 11F (3900-4000) | CAG GCT TGA CCA TTT TTC TAT C |
| 20 | KFD 11R (4026-4007) | GAT AGA AAA ATG GTC AAG CCA G |
| 22 | KFD 12F (4500-4600) | CTG AAC TTG TCT TCT CTG GAC |
| 23 | KFD 12R (4598-4578) | GTC CAG AGA AGA CAA GTT CAG |
| 24 | KFD 13F (5000-5100) | CAG GCT GGA TGT CAA AAG G |
| 25 | KFD 13R (5151-5133) | CCT TTT GAC ATC CAG CCT G |
| 26 | KFD 14F (5500-5600) | CTT TGA CTG GAT AAC AGA GTA TG |
| 27 | KFD 14R (5605-5583) | CAT ACT CTG TTA TCC AGT CAA AG |
| 28 | KFD 15F (6100-6200) | GAG GAG AAA AGG AAA CAC TTC |
| 29 | KFD 15R (6129-6109) | GAA GTG TTT CCT TTT CTC CTC |
| 30 | KFD 16F (6600-6700) | GGT GCT TTG TGG CAA GAA CTT |
| 31 | KFD 16R (6613-6593) | AAG TTC TTG CCA CAA AGC ACC |
| 32 | KFD 17F (7000-7100) | CAG ACA AAA ATA CAA CAA CTG GTG |
| 33 | KFD 17R (7115-7092) | CAC CAG TTG TTG TAT TTT TGT CTG |
| 34 | KFD 18F (7552-7571) | CTG AGA CAC TCT GGA CAA TG |
| 35 | KFD 18R (7491-7472) | CAT TGT CCA GAG TGT CTC AG |
| 36 | KFD 19F (7800-7900) | CCT TGA AAG GTG TGG TTG TTG |
| 37 | KFD 19R (7825-7805) | CAA CAA CCT CAC CTT TCA AGG |
| 38 | KFD 20F (8200-8300) | GAG ATG TAC TTC TCC ACT GC |
| 39 | KFD 20R (8264-8245) | GCA GTG GAG AAG TAC ATC TC |
| 40 | KFD 21F (8650-8750) | CAG GTG GGA ACC AAG ATC AT |
| 41 | KFD 21R (8711-8692) | ATG ATC TTG GTT CCC ACC TG |
| 42 | KFD 22F (9000-9100) | GAA TTT GAG GCA CTG GGT TTC |
| 43 | KFD 22R (9066-9046) | GAA ACC CAG TGC CTC AAA TTC |
| 44 | KFD 23F (9400-9500) | CTC ACG AAC ATC AAG GTG C |
| 45 | KFD 23R (9448-9430) | GCA CCT TGA TGT TCG TGA G |
| 46 | KFD 24F (9800-9900) | CAG CAT GTC TCT CAA AGG C |
| 47 | KFD 24R (9863-9845) | GCC TTT GAG AGA CAT GCT G |
| 48 | KFD 25F (10140-10240) | CAG TGG AGA AAG TGA GGA G |
| 49 | KFD 25R (10208-10190) | CTC CTC ACT TTC TCC ACT G |
| 50 | KFD 26R (10491-10473) | AAA TGG TGT GCT GCC ACA G |

**Supplementary Table S7: Primer combinations and positions in the reference KFDV genome**

| **Sr.No.** | **RT-PCR Primer Pairs** | **Size Of PCR Product** | **Position in the**  **KFDV Genome** |
| --- | --- | --- | --- |
| 1 | 1F+5R | 1.2 Kb | 132 – 1328 |
| 2 | 4F+7R | 1.2 Kb | 921 – 2140 |
| 3 | 5F+ 8R | 1.4 Kb | 1310 – 2690 |
| 4 | 7F + 9R | 1.1 Kb | 2120 – 3250 |
| 5 | 8F + 10R | 1.1 Kb | 2673 – 3791 |
| 6 | 9F+11R | 824 bp | 3230 – 4054 |
| 7 | 9F+12R | 1.4 Kb | 3230 – 4626 |
| 8 | 11F+13R | 1.2 Kb | 4033 – 5179 |
| 9 | 12F+14R | 1.51 Kb | 4606 – 5685 |
| 10 | 13F+15R | 1.1 Kb | 5161-6209 |
| 11 | 14F+16R | 1.1 Kb | 5683-6673 |
| 12 | 15F+18R | 1.4 Kb | 6189-7575 |
| 13 | 17F+19R | 813 bp | 7092-7905 |
| 14 | 18F+21R | 1.1Kb | 7552-8791 |
| 15 | 20F+23R | 1.2 Kb | 8325-9528 |
| 16 | 21F+24R | 1.2 Kb | 8772-9943 |
| 17 | 22F+26R | 1.5 Kb | 9126-10491 |
| 18 | 24F+26R | 566 bp | 9925-10491 |

**Supplementary Table S8**: Details of the additional 28 E-genes from KFDV isolates obtained from GenBank.

| **Sr. no.** | **ID. No.** | **Year** | **Location** | **Isolation Source (host)** | **Passage No.** | **Sample type** | **Accession No** |
| --- | --- | --- | --- | --- | --- | --- | --- |
| 1 | G11338 | 1957 | India: Barasi, Karnataka | *Haemaphysalis spinigera* | P-6 | M.Br. | JF416959 |
| 2 | W-377 | 1957 | India: Baragi, Karnataka | *S. entellus* serum | P-4 | M.Br. | JF416960 |
| 3 | G11333 | 1957 | India: Barasi, Karnataka | *Haemaphysalis spinigera* | P-3 | M.Br. | EU293276 |
| 4 | W3399 | 1958 | India: Hessare, Karnataka | *S. entellus* Lung | P-5 | M.Br. | EU293284 |
| 5 | P20924 | 1959 | India: Mullukere, Karnataka | *H. sapiens* serum | P-6 | M.Br. | EU293280 |
| 6 | W6178 | 1959 | India: Kannahalli, Karnataka | *S. entellus* brain | P-3 | M.Br. | EU293287 |
| 7 | 601011 | 1960 | India: Chikkasakuna, Karnataka | *H. sapiens* serum | P-4 | M.Br. | EU293242 |
| 8 | 611661 | 1961 | India: Sagar, Karnataka | *Haemaphysalis turturis* | P-5 | M.Br. | EU293244 |
| 9 | 612057 | 1961 | India: Barur, Karnataka | *Rattus rattus wroughtoni* spleen | P-6 | M.Br. | EU293245 |
| 10 | 623969 | 1962 | India: Karnataka state | *H. sapiens* | P-4 | M.Br. | EU293246 |
| 11 | 64244 | 1964 | India: Balagodu, Karnataka | *Ixodes petauristae* | P-3 | M.Br. | EU293254 |
| 12 | 652 | 1965 | India: Kangodu II, Karnataka | *Haemaphysalis wellingtoni* | P-5 | M.Br. | EU293257 |
| 13 | 651521 | 1965 | India: Karnataka state | *H. sapiens* | P-2 | M.Br. | EU293256 |
| 14 | 664518 | 1966 | India: Kondagalale I, Karnataka | *H. turturis* | P-2 | M.Br. | EU293261 |
| 15 | 68142 | 1968 | India:Holagalale, Karnataka | *Presbytis entellus* serum | P-4 | M.Br. | EU293266 |
| 16 | 716810 | 1971 | India:Gunjnur, Karnataka | *H. spinigera* | P-5 | M.Br. | EU293272 |
| 17 | 72166 | 1972 | India:Gadgeri-Sirsi, Karnataka | *Haemaphysalis kyasanurensis* | P-6 | M.Br. | EU293273 |
| 18 | NIV135724 | 2013 | India: Kerala state | *H. sapiens* | Clinical sample | Human serum samples | KY779866 |
| 19 | NIV146034 | 2014 | India: Mallapuram, Kerala | *H. sapiens* | Clinical sample | Human serum samples | KP315947 |
| 20 | MCL-15-T-338 | 2015 | India: Kerala state | *Haemaphysalis sps.* | Tick homogenate | Tick samples | KY779867 |
| 21 | NIV164187 | 2016 | India: Belgaun, Karnataka | *H. sapiens* | P-1 | M.Br. | MF186840 |
| 22 | NIV166149 | 2016 | India: Caranzalem, Goa | *H. sapiens* | P-1 | M.Br. | MF186844 |
| 23 | NIV162235 | 2016 | India: Sindhudurg, Maharashtra | *H. sapiens* | P-2 | BHK-21 TCF | MF186838 |
| 24 | NIV16848 | 2016 | India: Sindhudurg, Maharashtra | *H. sapiens* | P-2 | M.Br. | MF186845 |
| 25 | NIV16827 | 2016 | India: Sindhudurg, Maharashtra | *H. sapiens* | P-1 | M.Br. | MF186846 |
| 26 | NIVAN163939 | 2016 | India: Caranzalem, Goa | *Semnopithecus entellus* | P-2 | M.Br. | MF186841 |
| 27 | NIVAN161406 | 2016 | India: Sindhudurg, Maharashtra | *Macaca radiata* | P-1 | M.Br. | MF186842 |
| 28 | NIVAN161919 | 2016 | India: Goa state | *Semnopithecus entellus* | P-1 | M.Br. | MF186843 |
